# Responses to Salinity in Four *Plantago* Species from Tunisia

**DOI:** 10.3390/plants10071392

**Published:** 2021-07-07

**Authors:** Hela Belhaj Ltaeif, Anis Sakhraoui, Sara González-Orenga, Anbu Landa Faz, Monica Boscaiu, Oscar Vicente, Slim Rouz

**Affiliations:** 1 Institute for the Conservation and Improvement of Valencian Agrodiversity (COMAV), Universitat Politècnica de València, Camino de Vera s/n, 46022 Valencia, Spain; belhajhela1@gmail.com (H.B.L.); sagonor@doctor.upv.es (S.G.-O.); anbu.landafaz@cinvestav.mx (A.L.F.); 2 National Agronomy Institute–Tunis, University of Carthage, Mahrajène, 1082 Tunis, Tunisia; 3 Laboratory of Agricultural Production Systems and Sustainable Development (LR03AGR02), Department of Agricultural Production, Agricultural High School of Mograne, University of Carthage, 1121 Mograne-Zaghouan, Tunisia; anis.sakhraoui@esakef.u-jendouba.tn (A.S.); slim.rouz@esamg.u-carthage.tn (S.R.); 4 Agricultural High School of Kef, Jendouba University, 7119 Le Kef, Tunisia; 5 Departamento de Biología Vegetal y Ecología, Universidad de Sevilla, Apartado 1095, 41080 Sevilla, Spain; 6 Mediterranean Agroforestry Institute (IAM), Universitat Politècnica de València, Camino de Vera s/n, 46022 Valencia, Spain; mobosnea@eaf.upv.es; 7 Center for Research and Advanced Studies of the National Polytechnic Institute, Av. Instituto Politécnico Nacional No. 2508, Colonia San Pedro Zacatenco, C.P. 07360 Ciudad de México D.F., Mexico

**Keywords:** salt stress, halophytes, growth responses, ion accumulation, osmolytes, oxidative stress biomarkers, antioxidants

## Abstract

The genus *Plantago* is particularly interesting for studying the mechanisms of salt tolerance in plants, as it includes both halophytes and glycophytes, as well as species adapted to xeric environments. In this study, the salt stress responses of two halophytes, *P. crassifolia* and *P. coronopus,* were compared with those of two glycophytes, *P. ovata* and *P. afra*. Plants obtained by seed germination of the four species, collected in different regions of Tunisia, were subjected to increasing salinity treatments for one month under greenhouse conditions. Morphological traits and biochemical parameters, such as ion accumulation and the leaf contents of photosynthetic pigments, osmolytes, oxidative stress markers and antioxidant metabolites, were measured after the treatments. Salt-induced growth inhibition was more pronounced in *P. afra*, and only plants subjected to the lowest applied NaCl concentration (200 mM) survived until the end of the treatments. The biochemical responses were different in the two groups of plants; the halophytes accumulated higher Na^+^ and proline concentrations, whereas MDA levels in their leaves decreased, indicating a lower level of oxidative stress. Overall, the results showed that *P. coronopus* and *P. crassifolia* are the most tolerant to salt stress, and *P. afra* is the most susceptible of the four species. *Plantago ovata* is also quite resistant, apparently by using specific mechanisms of tolerance that are more efficient than in the halophytes, such as a less pronounced inhibition of photosynthesis, the accumulation of higher levels of Cl^−^ ions in the leaves, or the activation of K^+^ uptake and transport to the aerial part under high salinity conditions.

## 1. Introduction

Global agricultural yields and food production are negatively affected by different environmental stress factors, especially drought and salinity [1,2]. These stressors inhibit plant growth and reproductive development, causing significant reductions in crop productivity and may even compromise yield entirely. Salinity is already affecting 25–30% of total cultivated land and 33% of irrigated land [3], although some estimates increase this percentage to more than 50% [4]. This situation is predicted to worsen shortly due to the consequences of climate change [5], as more cultivated areas will depend on irrigation and lower-quality water will be used, triggering an increase in the rate of secondary soil salinisation [6]. Salinity impairs plant growth and development due to its two components, osmotic stress and ion toxicity, inhibiting plant growth and cellular functions and, eventually, causing plant death [7,8,9,10]. Plants exposed to salt stress show morphological, physiological, metabolic, and molecular changes reflected, for example, in a delayed or completely inhibited seed germination, high seedling mortality [11] or a general inhibition of photosynthesis and growth [2,8,12]. Although most plants are glycophytes, susceptible to salinity, there is a small group of ca. 1500 species from different genera and families that are halophytes, which can survive and complete their life cycle on saline soils [13]. 

The genus *Plantago* L. (Plantaginaceae family) includes more than 250 annual and perennial herbs and subshrubs, distributed worldwide, except for tropical rainforest and the Antarctic. Some *Plantago* species are cosmopolitan, others have more limited geographical ranges, but the genus also includes local endemics [14,15]. There are many interesting aspects related to the taxonomy and evolutionary trends of this genus [16,17], but also concerning salt stress physiology and biochemistry, given that it includes several well-known halophytes [18,19,20,21].

*Plantago coronopus* L. is an annual or biennial species that ranges from North Africa and the Iberian Peninsula to SE Asia, reaching northern Europe through a narrow strip along the Atlantic coast [22,23]. It grows in different types of littoral and inland habitats, such as sand dunes, saline grasslands, salt marshes, scrublands, or human-disturbed areas [23], tolerating saline soils [24]. It is considered as a potential cash crop [24], an edible plant with nutraceutical [25] and antioxidant properties [26]. 

*Plantago crassifolia* Forsk. is a perennial species present only in the Mediterranean region. South African populations, previously ascribed to this species, are now considered as *P. carnosa* Lam, based on the analysis of the internal transcribed spacer (ITS) region of rRNA genes [27]. *Plantago crassifolia* is a typical halophyte, growing exclusively in saline habitats with moderate soil salinity and occupying interdune depressions and salt marsh edges [21,28]. It is reported as palatable fodder [29]. The two species, *P. coronopus* and *P. crassifolia,* are taxonomically related, belonging to the subgenus *Coronopus* (Lam. and DC.) Rahn, section *Coronopus* Lam [30]. 

*Plantago ovata* Forssk. is an annual or short-lived perennial species, ranging from the Canary Islands and SE Iberian Peninsula, across northern Africa, to India [31]. It was considered introduced in North America in the 18th century, but a molecular clock based on ITS and chloroplast DNA analysis dates a much earlier, non-anthropogenic introduction from the Old World, 200,000–650,000 years ago [32]. The species grows in dry areas on wasteland, annual pastures, almost always on somewhat nitrified soils, indifferent to soil pH, but has also been found, occasionally, in moderately saline soils [31]; in North America, it is present only in desert and Mediterranean habitats [33]. Due to the laxative properties of the seed mucilage, *P. ovata* is a well-known medicinal plant cultivated in many countries, with India as the leading producer [34]. 

*Plantago afra* L. (syn. *P. psyllium* L.) is an annual species with a wide geographic distribution, from the Canary Islands and the Iberian Peninsula, along the Mediterranean region, to Pakistan. It grows in annual grasslands, roadsides, and crop fields in semi-arid and arid areas [31]. Like *P. ovata*, it has medicinal applications and is cultivated in India, Pakistan, and Iran. The seed husks of *P. ovata* and *P. afra* are known by the name ’psyllium‘ and are a popular mild laxative used to relieve chronic constipation, bowel cancer and gastrointestinal irritation. Psyllium is also used as a dietary source of fibre to treat obesity and cholesterol reduction or as an antitussive and anti-inflammatory [35]. The two species belong to the subgenus *Psyllium* (Juss.) Harms and Reich; *P. ovata* is classified in the section *Albicans* Barnéoud, and *P. afra* in the section *Psylliu*m (Juss.) Lam and DC [30].

This work aimed to compare the responses to salt stress in two typical halophytes of the genus *Plantago*, *P. crassifolia* and *P. coronopus*, and two other congeneric species, *P. ovata* and *P. afra*, more adapted to xeric environments. Although the resistance to salinity has been evaluated in many plants, comparative analyses in genetically related species adapted to different natural habitats are not so commonly performed and can provide insights into the most relevant mechanisms of salt tolerance in a particular genus. To address this question, inhibition of growth in the presence of increasing salt concentrations, up to 800 mM NaCl, was evaluated in plants of the four species as the most reliable parameter to establish their relative degree of tolerance. Then, these growth responses were correlated with the salt-induced changes in the levels of biochemical markers associated with specific stress response pathways, namely, monovalent ions (Na^+^, Cl^−^, K^+^), specific osmolytes (proline and total soluble sugars), oxidative stress biomarkers (malondialdehyde, H_2_O_2_) and antioxidant compounds (phenolics and flavonoids). 

## 2. Results

In this study, morphological, physiological, and biochemical traits were measured to analyse the impact of salinity stress treatments on four *Plantago* species (*P. coronopus, P. crassifolia, P. ovata* and *P. afra*). 

A two-way ANOVA was performed to assess the effects of the factors ‘species’, ‘treatment’, and their interactions, on all measured parameters (Table 1). The 21 traits analysed displayed significant differences for the ‘species’ effect. Differences between treatments were also significant for all measured parameters, except K^+^ levels in leaves, whereas leaf fresh weight was the only non-significant trait regarding the interaction of species and treatments. 

### 2.1. Substrate Analysis 

The electrical conductivity of the substrate in the pots (EC_1:5_) at the end of the salt treatments increased in parallel with the NaCl concentration in the irrigation water, with significant differences between treatments (Table 2). EC reached the highest values in the pots watered with 800 mM NaCl, 9.57 dS m^−1^ for pots with *P. coronopus* and 9.45 dS m^−1^ for *P. crassifolia*. No significant differences were observed between different species for each salt concentration tested. EC was not determined (n.d.) for those treatments that resulted in the death of the plants, 800 mM NaCl for *P. ovata* or 400 mM and higher salt concentrations in the case of *P. afra* (Table 2).

### 2.2. Effects of Salt Stress on Plant Growth

**Figure 1:**
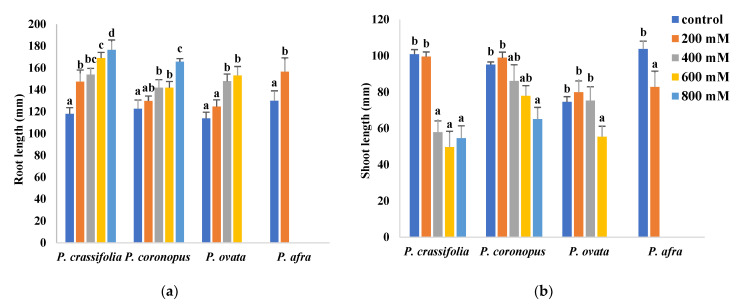
Root length (**a**) and shoot length (**b**) of the four selected *Plantago* species after four weeks of treatment with the indicated NaCl concentrations. The values shown are means ± SE (*n* = 5). For each species, different letters over the bars indicate significant differences between treatments, according to the Tukey test (*p* < 0.5).

*Plantago ovata* plants did not survive the four-week treatment with the highest salt concentration applied, 800 mM NaCl; therefore, samples from this treatment were not collected. In the case of the more salt-sensitive *P. afra*, only data from the control and the 200 mM NaCl treatments were obtained since the plants could not withstand 400 mM NaCl or higher salinities and died within the first two or three weeks of treatment. Although the halophytes, *P. crassifolia* and *P. coronopus*, survived all treatments without showing any apparent wilting symptoms, even in the presence of 800 mM NaCl, salt stress affected growth in all four species (Figure 1). For example, in all cases, root length increased in parallel with increasing external salinity (Figure 1a). Stimulation of root growth in response to salt stress seems to mimic the behaviour of the plants in nature, where high salt concentrations may induce roots to grow, searching for soil layers with lower salinity.

All other measured parameters showed growth inhibition in response to the salt treatments in the four *Plantago* species. The experimental data also revealed that *P. ovata* is relatively resistant to salinity, even though it is not considered a typical halophytic species since it is not present in highly saline natural habitats.

Measurements of shoot length in plants treated with different salt concentrations allowed estimating the relative salt tolerance of the investigated species. According to this criterion, *P. coronopus* appears to be the most tolerant, with a statistically significant reduction of shoot length, compared to the non-stressed control, observed only in the presence of 800 mM NaCl. In *P. ovata*, no reduction was detected in plants subjected to the 200 and 400 mM NaCl treatments, whereas a significant decrease in shoot length was observed in *P. crassifolia* plants treated with 400 and higher NaCl concentrations. The most salt-sensitive species, *P. afra*, already showed inhibition of shoot growth at 200 mM NaCl, the only salt concentration that allowed survival of the plants under the conditions used in the experiments (Figure 1b). 

**Figure 2:**
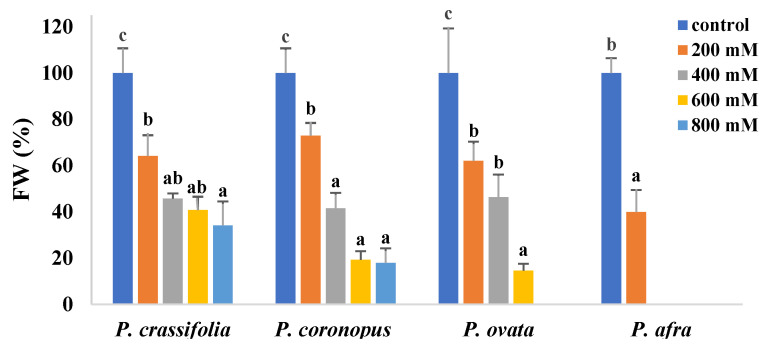
Fresh weight (FW) reduction of the aerial part of the plants in the four selected *Plantago* species after four weeks of treatment with the indicated NaCl concentrations. For each species, different letters over the bars indicate significant differences between treatments, according to the Tukey test (*p* < 0.5). Values are shown as percentages of the FW of the corresponding controls, taken as 100%.

Determination of the fresh weight of the aerial part of the plants confirmed the highest salt sensitivity of *P. afra*, for which a FW decrease of about 60% of the control was calculated in the presence of 200 mM NaCl. Growth inhibition between 200 and 600 mM NaCl followed a similar pattern for *P. coronopus* and *P. ovata*. The relative FW reduction at high salinities was comparatively lower in *P. crassifolia*, probably due to less water loss, given the succulent leaves of this species (Figure 2). Values in Figure 2 are shown as percentages of the corresponding non-stressed controls to better compare the four species, which have slightly different sizes.

**Figure 3:**
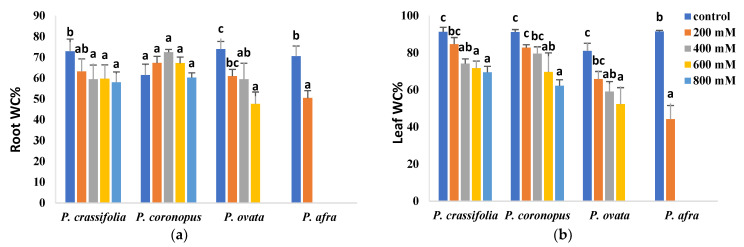
Water content percentage in roots (**a**) and leaves (**b**) in the four selected *Plantago* species, after four weeks of treatment with the indicated NaCl concentrations. The values shown are means ± SE (*n* = 5). For each species, different letters over the bars indicate significant differences between treatments, according to the Tukey test (*p* < 0.5).

The relative salt tolerance of the four *Plantago* species correlated with their resistance to salt-induced dehydration in roots and leaves (Figure 3). In roots, non-significant or slight reductions in water contents were observed for *P. crassifolia* and *P. coronopus*, which kept high, and similar, values even in the presence of 800 mM NaCl; root dehydration was relatively higher in *P. ovata* and, especially, in the least tolerant *P. afra* (Figure 3a). Salt-induced dehydration was more pronounced in the leaves than in the roots, although maintaining the same general pattern. Leaf water loss was slightly lower in the succulent *P. crassifolia* than in *P. coronopus* but substantially lower in the two halophytes than in the other two species (Figure 3b).

### 2.3. Effects of Salt Stress on Photosynthetic Pigment Levels

**Figure 4:**
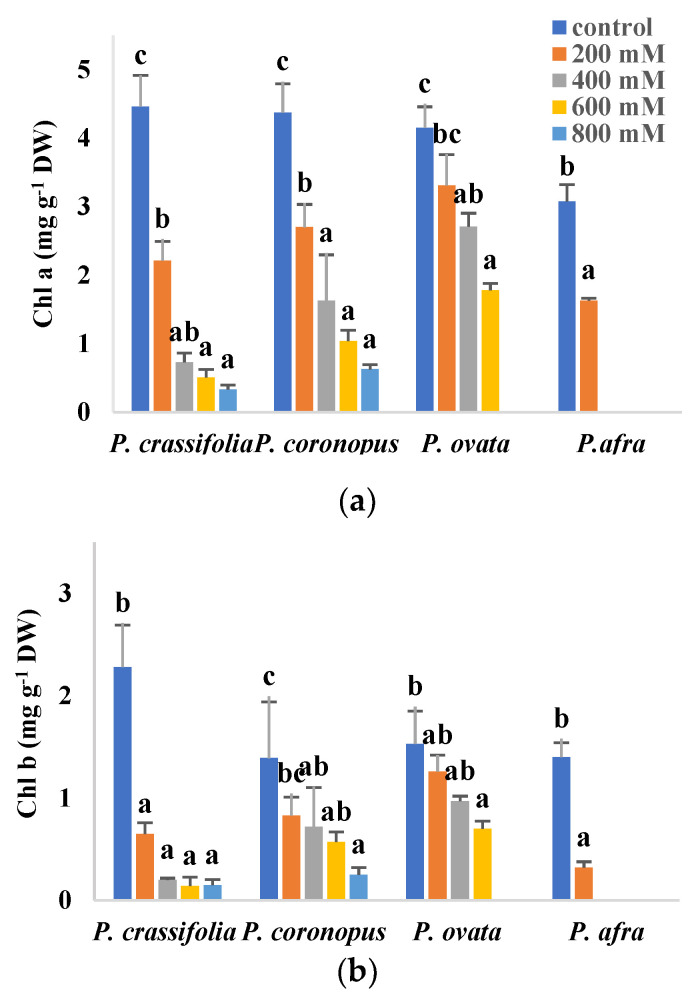

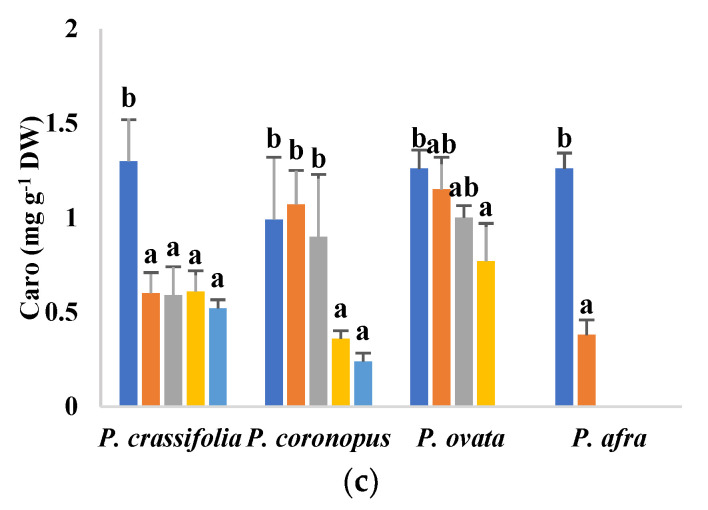
Chlorophyll a (Chl a) (**a**), chlorophyll b (Chl b) (**b**) and carotenoids (Caro) (**c**) contents in leaves of the four selected *Plantago* species, after four weeks of treatment with the indicated NaCl concentrations. The values shown are means ± SE (*n* = 5). For each species, different letters over the bars indicate significant differences between treatments, according to the Tukey test (*p* < 0.5).

Inhibition of photosynthesis, partly due to degradation of photosynthetic pigments, is a general response of plants to abiotic stress. In the present study, a significant, concentration-dependent decrease in chlorophylls a and b and carotenoid contents, with respect to the non-stressed controls, has been observed in plants of the four selected *Plantago* species subjected to increasing salt treatments (Figure 4). In the presence of 200 mM NaCl, the lowest values of the three pigments were measured in *P. afra*, the most salt-sensitive species, whereas no significant reduction was observed in *P. ovata* or, for Chl b and carotenoids, in *P. coronopus*. At higher salinities, *P. ovata* and the two halophytes showed similar qualitative patterns of variation, with the most substantial reduction in pigment contents generally detected in *P. crassifolia* and the least pronounced in *P. ovata* (Figure 4).

### 2.4. Ion Accumulation

**Figure 5:**
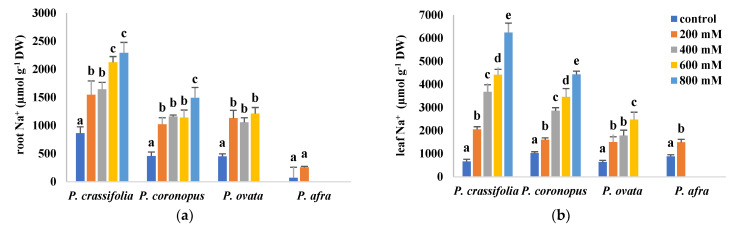

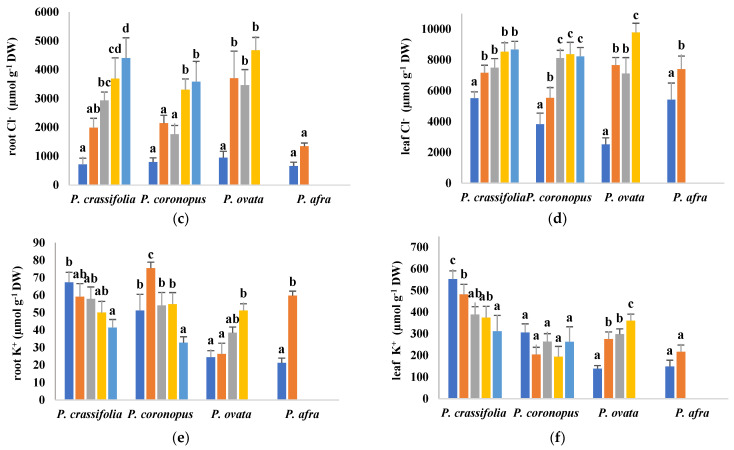
Root (**a**,**c**,**e**) and leaf (**b**,**d**,**f**) contents of sodium (Na^+^), (**a**,**b**), chloride (Cl^−^) (**c**,**d**) and potassium (K^+^) (**e**,**f**) in plants of the four selected *Plantago* species, after four weeks of treatment with the indicated NaCl concentrations. The values shown are means ± SE (*n* = 5). For each species, different letters over the bars indicate significant differences between treatments, according to the Tukey test (*p* < 0.5).

In the four investigated *Plantago* species, Na^+^ and Cl^−^ concentrations increased in the roots and leaves of the plants, roughly in parallel with the increasing NaCl concentration in the irrigation water (Figure 5). For each species and specific salt treatment, the concentrations of Cl^- ^ were consistently higher than those of Na^+^ in both organs, and those of both ions were higher in the leaves than in the roots (Figure 5a–d), indicating the existence of mechanisms for their active transport to the aboveground organs. There were, however, differences in the accumulation patterns of the two ions. In roots, Na^+^ contents were highest at all tested salinities (including the non-stressed controls), in *P. crassifolia*, and lowest in *P. afra*, whereas *P. coronopus* and *P. ovata* showed intermediate and similar values (Figure 5a). The same pattern of Na^+^ accumulation was observed in the leaves, except that the absolute concentrations reached, at the same external salinity, were higher in *P. coronopus* than in *P. ovata*. Note that a 10-fold increase in Na^+^ content was observed in the leaves of non-stressed *P. afra* plants with respect to the values measured in control roots (Figure 5b).

Regarding Cl^−^ in roots, apart from the lowest levels found in *P. afra*, its accumulation patterns were somewhat different from those of Na^+^, with *P. ovata* showing higher concentrations than *P. crassifolia* and *P. coronopus*, at the same salinity level (Figure 5c). Similar behaviour was observed in the leaves for the latter three species, whereas, contrary to roots, *P. afra* accumulated Cl^−^ to the same or even higher levels than the other species (Figure 5d). Nevertheless, the most striking feature was the huge Cl^−^ concentration determined in the leaves of control plants, especially in *P. crassifolia* and *P. afra*, not only in relation to the root contents (about eight-fold higher), but also in absolute terms (over 5 mmol g^−1^ DW) (Figure 5d).

Despite quantitative differences, Na^+^ and Cl^−^ concentrations varied in the same way, qualitatively, in the four *Plantago* species in response to the salt treatments, always increasing with increasing salinity. However, the salt-induced changes in K^+^ contents differed in the different taxa (Figure 5e, f). In *P. crassifolia*, K^+^ levels decreased progressively, in roots and leaves, roughly in parallel with the increase of NaCl concentration in the irrigation water, whereas no significant variation was observed, in general, in *P. coronopus*, except for a significant reduction in roots in the presence of 800 mM NaCl. On the contrary, in *P. ovata* and *P. afra,* K^+^ contents increased in response to the salt stress treatments. It should also be mentioned that, as for the other ions, K^+^ levels were higher (five to ten-fold) in leaves than in roots in all four species (Figure 5e,f).

### 2.5. Salt Stress Effect on Osmolyte Contents

**Figure 6:**
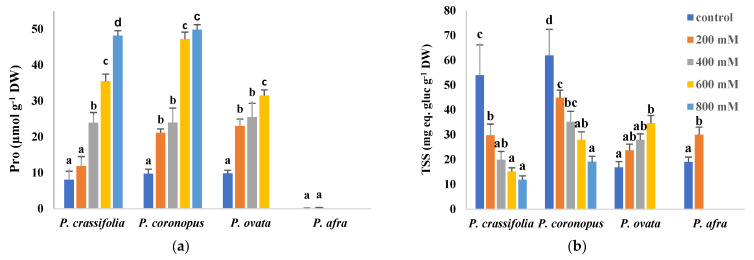
Leaf contents of proline (Pro) (**a**) and total soluble sugars (TSS) (**b**) in the four selected *Plantago* species, after four weeks of treatment with the indicated NaCl concentrations. The values shown are means ± SE (*n* = 5). For each species, different letters over the bars indicate significant differences between treatments, according to the Tukey test (*p* < 0.5). gluc: glucose.

Proline (Pro), one of the most common plant osmolytes, accumulated in response to the salt treatments in the leaves of the two halophytes, *P. crassifolia* and *P. coronopus*. Pro reached maximum levels of about 50 µmol g^−1^ DW in the presence of 800 mM NaCl, representing an increase of five to six-fold over control values. Leaf Pro concentrations also increased in *P. ovata*, but only up to ~30 µmol g^−1^ DW at the highest concentration tested, 600 mM NaCl. In the most salt-sensitive species, *P. afra*, Pro remained extremely low, below one µmol g^−1^ DW (Figure 6a). However, total soluble sugars (TSS) contents showed different patterns of variation, increasing with increasing external salinity only in *P. ovata* and *P. afra* but decreasing in the halophytes (Figure 6b).

### 2.6. Oxidative Stress Biochemical Markers

**Figure 7:**
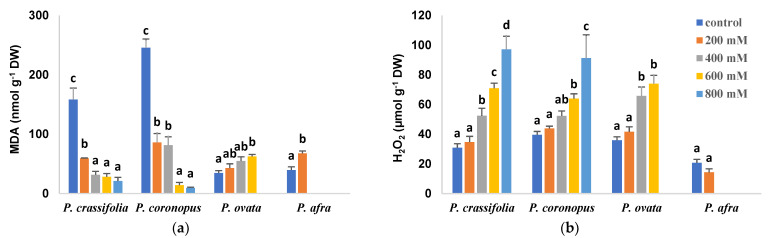
Leaf contents of malondialdehyde (MDA) (**a**) and hydrogen peroxide (H_2_O_2_) (**b**) in the four selected *Plantago* species, after four weeks of treatment with the indicated NaCl concentrations. The values shown are means ± SE (*n* = 5). For each species, different letters over the bars indicate significant differences between treatments, according to the Tukey test (*p* < 0.5).

Salt-induced changes in the leaf levels of malondialdehyde (MDA) followed a similar pattern to those of TSS, increasing in parallel to the NaCl concentrations in the irrigation water in *P. ovata* and *P. afra* and progressively decreasing in *P. crassifolia* and *P. coronopus* (Figure 7a). On the other hand, hydrogen peroxide leaf contents did not vary significantly in *P. afra* treated with 200 mM NaCl and increased significantly, in a concentration-dependent manner, in salt-treated plants of the other three species (Figure 7b).

### 2.7. Antioxidant Compounds

**Figure 8:**
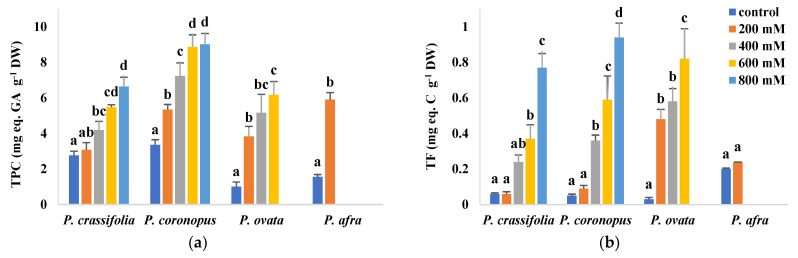
Leaf contents of total phenolic compounds (TPC) (**a**) and total flavonoids (TF) (**b**) in the four selected *Plantago* species, after four weeks of treatment with the indicated NaCl concentrations. The values shown are means ± SE (*n* = 5). For each species, different letters over the bars indicate significant differences between treatments, according to the Tukey test (*p* < 0.5). GA: gallic acid; C. catechin.

The leaf contents of total phenolic compounds (TPC) and total flavonoids (TF), as representative examples of non-enzymatic antioxidants, were measured in plants of the four investigated *Plantago* species (Figure 8). TPC levels increased in the four taxa in response to rising salinity, reaching the highest values in *P. coronopus* (9 mg equivalent of gallic acid per gram DW) and *P. crassifolia* (about 6.6 mg eq. GA g^−1^ DW), in the presence of 800 mM NaCl, which represent relative increases over the control, non-stressed plants of 2.7 and 2.4-fold, respectively. In *P. ovata* and *P. afra*, control TPC concentrations were lower than in their halophytic counterparts and, therefore, these species showed larger relative increases in response to salt stress (Figure 8a). TF contents also increased significantly with rising salinity, except for *P. afra*. For each NaCl concentration, both absolute TF levels and relative increases over control values were highest for *P. ovata* and lowest in *P. crassifolia* (Figure 8b).

### 2.8. Principal Component Analysis

**Figure 9:**
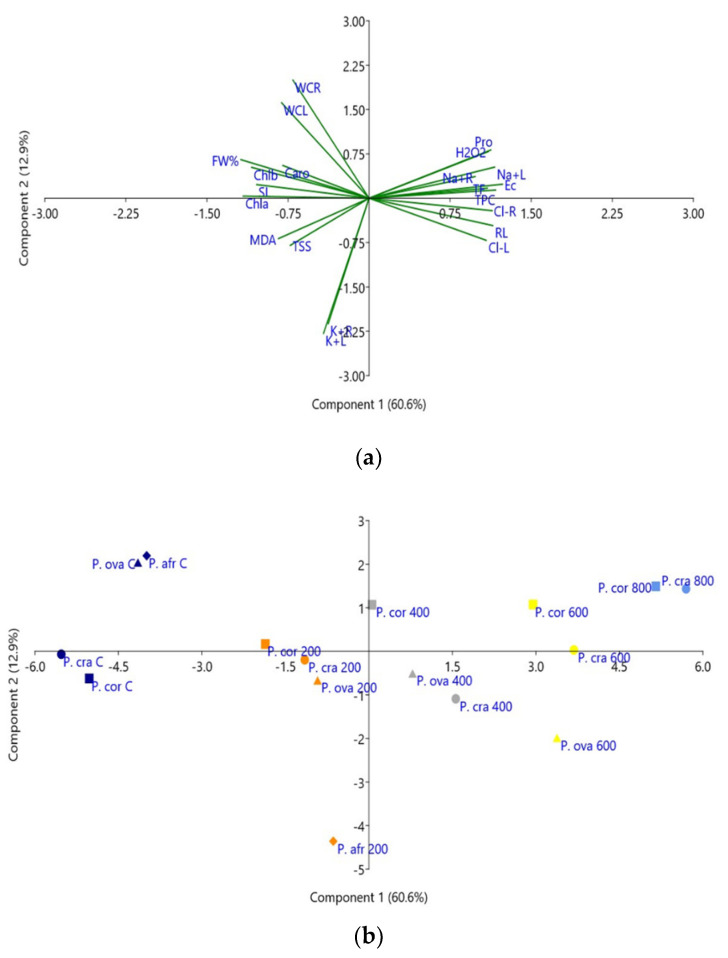
Loading plot of the principal component analysis (PCA) (**a**), and scatterplot of the PCA score (**b**), including all analysed traits in *P. coronopus* (P. cor), *P. crassifolia* (P. cra), *P. ovata* (P. ova) and *P. afra* (P. afr) subjected to treatments with 0 (control, C), 200, 400, 600 and 800 mM NaCl, for four weeks. The first and second principal components accounted for 60.6% and 12.9% of the total variation, respectively. Abbreviations: electrical conductivity of substrate (EC); root length (RL); stem length (SL); root water content (RWC); leaf water content (LWC); fresh weight of leaves (FW%); chlorophyll a (Chla) chlorophyll b (Chlb); total carotenoids (Caro); sodium content in leaves and roots (Na+L; Na+R); chloride content in leaves and roots (Cl-L; Cl-R); potassium content in leaves and roots (K+L; K+R), total soluble sugars (TSS), malondialdehyde (MDA), hydrogen peroxide (H2O2); proline (Pro); total phenolic compounds (TPC) and flavonoids (TF).

A Principal Component Analysis (PCA) was performed, including all variables measured in the four *Plantago* species, both growth parameters and the biochemical stress markers (Figure 9). Five components with an eigenvalue higher than one were detected. The first component (*X*-axis), explaining 60.6% of the total variance, was positively correlated with the electrical conductivity (EC) of the substrate; that is, with soil salinity (Figure 9a). Consequently, all variables that increased with increasing salinity, root length, Na^+^ and Cl^−^ contents in roots and leaves, Pro, H_2_O_2_ and antioxidant compounds, were also positively correlated with the first component. On the other hand, a negative correlation was found with the rest of the growth parameters (in agreement with the observed salt-induced inhibition of growth) and with photosynthetic pigments, MDA and TSS, which generally decreased in response to the salt treatments. The second component, explaining an additional 12.9% of the total variability, was mostly correlated, negatively, with K^+^ levels in roots and leaves (Figure 9a).

The scatterplot of the projected variables (Figure 9b) allowed a good separation of the salt treatments along the *X*-axis, from the non-stressed plants to those subjected to the highest salinity levels. A clear separation was also observed under control conditions between *P. crassifolia* and *P. coronopus* on the one side and *P. ovata* and *P. afra* on the other. Moreover, the response of *P. afra* to salt stress at 200 mM NaCl, the only treatment allowing survival of the plants of this less tolerant species, clearly differed from that of the other three taxa, in agreement with the relatively high salt tolerance of *P. ovata*, similar to that of the halophytes (Figure 9b).

## 3. Discussion

The four *Plantago* species analysed in the present work can be separated into two taxonomic groups; *P. crassifolia* and *P. coronopus* belong to the subgenus *Coronopus*, and *P. ovata* and *P. afra* are included in the subgenus *Psyllium*. Moreover, the first two species are defined as halophytes, whereas the other two are considered glycophytes. These differences are reflected in the positions of the four species in the scatterplot of the PCA score. Nevertheless, the present work results indicated that, although *P. afra* is indeed sensitive to salinity, *P. ovata* is quite salt-tolerant, apparently because it can use some specific tolerance mechanisms more efficiently than the halophytes.

As established for many different plant species, salt stress induces changes in root system morphology, growth rate and reproductive traits in *Plantago* [21,36,37]. However, the relative survival thresholds and the quantitative assessment of stress-induced growth inhibition are probably the most objective criteria to rank taxonomically related species according to their tolerance to different environmental stressors such as salinity or drought [21,38,39,40]. Of the four analysed *Plantago* species, *P. afra* was the most susceptible to salt stress as the plants survived the one-month treatment only in the presence of 200 mM NaCl, the lowest salt concentration tested. A previous study also reported that growth of *P. afra* was significantly inhibited at salinities higher than 100 mM NaCl and the plants did not survive the concentration of 300 mM [35]. Of the remaining species, *P. crassifolia* and *P. coronopus* were the most stress-tolerant, as reported in previous studies [21,24], which agrees with their ecology. Plants of *P. ovata* were relatively more affected by salinity than the two halophytes; still, under our experimental conditions, they survived all salt treatments except that at very high salinity, 800 mM NaCl. Indeed, this species has been considered moderately salt-tolerant [41], although marked differences between genotypes have been reported in the responses to salinity [42,43].

Reduced plant growth is one of the first and most general responses to stress. Accordingly, a general effect of growth inhibition in the presence of salt has been observed in all four *Plantago* species. However, the plant roots significantly increased in length in parallel to increasing salinity. According to Neumann [44], a rapid root elongation may occur in salt-stressed plants due to the massive production of young cells by stimulation of root meristem divisions. A more extensive root system penetrates deeper soil layers to obtain water and nutrients; this implies a higher water uptake capacity in tolerant plants, allowing ion dilution to help avoid reaching toxic levels in the cytosol [45]. Similar results have been reported, for example, in salt-stressed *P. major* plants, where primary roots were longer at all salinity levels compared to control plants [18,36]. The reduction in growth parameters of the plants’ aerial parts, shoot length and fresh weight, and the level of leaf dehydration allowed us to establish the relative salt tolerance of the four species, as indicated above: *P. crassifoilia* ≅ *P. coronopus* > *P. ovata* >> *P. afra*.

Chlorophyll is a useful biochemical salt stress marker in plants, as high NaCl concentrations induce chlorophyll loss and necrosis of the leaves in many species [46,47]. Chlorophyll contents generally decrease in the presence of salt, often proportionally to the salt sensitivity of the plants, so that highly salt-tolerant halophytes may not show a reduction in chlorophyll levels under salinity conditions [12,48,49]. The decrease of photosynthetic pigments results from the inhibition of enzymes involved in chlorophyll biosynthesis and the fast breakdown of the pigments due to activation of chlorophyllase, responsible for chlorophyll degradation [50,51,52]. The selected *Plantago* species also showed this general pattern, a salt-induced, concentration-dependent decrease in photosynthetic pigment contents in response to the salt treatments. The reduction in the pigments’ concentrations roughly corresponded to the relative salt tolerance of the plants, except that *P. ovata* appeared to be less affected than the halophytes, with chlorophylls a and b, and carotenoid contents significantly lower than control values only observed at high salinities.

One of the most significant differences in the mechanisms of salt stress response between glycophytes and halophytes regards managing the toxic ions present on saline soils. Glycophtes and monocotyledonous halophytes generally rely on reducing ion uptake through their roots or blocking their transport to the leaves. On the other hand, dicotyledonous halophytes activate the transport of toxic ions to the aboveground plant organs to be used for osmotic adjustment, but sequester them in the vacuoles to avoid their deleterious effects in the cytosol [7,53]. Ion compartmentalisation in the vacuoles is an extremely efficient mechanism, cheaper in energy consumption terms than the synthesis of organic osmolytes for ensuring an increased osmotic potential [54]. In the present work, we show that ion concentrations were consistently higher in leaves than in roots, at each salt concentration tested and in the four *Plantago* species, supporting the existence of these mechanisms of active ion transport to the leaves. Nevertheless, the patterns of accumulation of Na^+^ and Cl^−^ differed quantitatively. Under salt stress, the glycophytes showed lower Na^+^ content in roots and leaves than the halophytes, with the highest absolute values measured in *P. crassifolia* leaves. These findings indicate that Na^+^ accumulation plays an essential role in the osmotic adjustment of halophytes of this genus subjected to high salinity conditions, as previously reported for these two species [21,24] and also for *P. maritima* [20,55,56,57].

Regarding Cl^−^ concentrations, the differences between species were not so pronounced as those of Na^+^, neither in roots nor in leaves; the most relevant difference was that, under the same salinity conditions, the glycophyte *P. ovata* accumulated Cl^−^ to higher concentrations than the halophytes *P. crassifolia* and *P. coronopus*. The extremely high Cl^−^ concentration measured in leaves of the control, non-stressed plants is also remarkable. These data point to a constitutive defence mechanism against salt stress based on the accumulation of high leaf concentrations of this anion, even under low salinity conditions.

Concerning K^+^, it is known that this ‘physiological cation’ plays an important role in plant growth and development, as well as in the maintenance of osmotic adjustment and cell turgor under stress [58]. A reduction of K^+^ contents is generally observed under salt stress conditions, resulting from competition between Na^+^ and K^+^ for the same binding sites in proteins, including ion transporters [59]. Therefore, maintenance, or even increases in leaf K^+^ levels in the presence of high Na^+^ concentrations may contribute significantly to salt tolerance mechanisms. Indeed, activation of K^+^ transport from roots to leaves at high salinities has been reported in some species, including glycophytes [60,61,62], and it is considered that salinity may enhance K^+^ transport through the vascular bundles [9,63]. The analysed *Plantago* species differed in the patterns of K^+^ transport and accumulation. The leaf K^+^ contents did not vary with increasing salinity in *P. coronopus*, whereas they increased significantly in salt-treated *P. ovata* plants, probably contributing to the tolerance of this species.

Plants accumulate compatible solutes such as proline (Pro) and soluble sugars (TSS) to contribute to osmotic adjustment, and as osmoprotectants, under different stress conditions [53]. The accumulation of these metabolites is one of the best-known responses of plants to changes in the external osmotic potential [7,64]. Many reports showed that sorbitol is the primary physiological osmolyte in species of the genus *Plantago*, both salt-tolerant [24,55,65] and salt susceptible [56]. However, the differences in absolute sorbitol levels accumulated in response to salt treatments do not explain the different salt tolerance of the investigated species. In several halophytes of this genus, activation of Pro biosynthesis has been observed at high external salinity [21,28,57,66,67]. Pro can be considered, therefore, as a secondary functional osmolyte in salt-tolerant *Plantago* species. Pro is one of the most common compatible solutes involved in stress tolerance mechanisms in plants, accumulated in large quantities under high salinity stress (and other stressful conditions) in many plant species [68,69,70]. Apart from playing a major role in osmotic adjustment, Pro can act as an enzyme protectant, free radical scavenger, cytosolic pH stabiliser for subcellular structures and cell redox balancer [71]. In the present study, the NaCl treatments induced a significant, concentration-dependent increase in the leaf Pro contents, especially in the halophytes, *P. crassifolia* and *P. coronopus*, at the highest concentrations tested, 600–800 mM NaCl, but also, to a lesser extent, in *P. ovata*, in agreement with previous reports [72]. On the other hand, extremely low Pro concentrations were measured in the salt-sensitive *P. afra*.

Soluble sugars have also been shown to accumulate in plants in response to abiotic stresses, contributing to osmotic adjustment and playing additional regulatory functions [73,74]. However, TSS do not seem to have any relevant role in salt tolerance mechanisms in the investigated *Plantago* species, although they showed different accumulation patterns in the halophytes and the glycophytes. Thus, TSS increased significantly in *P. ovata*, but only at the highest salt concentration tested, 600 mM NaCl, and in *P. afra* in the presence of 200 mM NaCl; however, the differences with respect to the corresponding controls are too small to have any important osmotic effect. Soluble sugar contents, on the contrary, decreased with increasing salinity in *P. crassifolia* and *P. coronopus*. These differences are probably due to a more pronounced salt-induced inhibition of photosynthesis in the halophytes, as revealed by the stronger reduction in pigment levels as compared with *P. ovata*.

Salt stress increases the production of reactive oxygen species (ROS), which, when in excess, have deleterious effects by oxidation of nucleic acids, lipids, and proteins, inducing severe dysfunctions and even cell death [75]. Malondialdehyde (MDA) is a product of membrane lipid peroxidation widely used as a biomarker of oxidative stress [76]. Leaf MDA contents decreased with increasing salinity in *P. crassifolia* and *P. coronopus*; on the contrary, they increased slightly in *P. ovata* and *P. afra*, with statistically significant differences with the corresponding control in the presence of 600 and 200 mM NaCl, respectively. This finding indicates that the halophytes are better protected from salt-induced oxidative damage of cell membranes, probably because of more efficient defence mechanisms based on toxic ion compartmentalisation and osmolyte (Pro) accumulation, as discussed above. Similar results were reported from a comparative study between the salt-tolerant *P. maritima* and the glycophyte *P. media*, with a decrease of MDA in the former and a significant increase in the latter species [77].

H_2_O_2_ is a ubiquitous, moderately reactive ROS with an essential role as a signalling molecule in stress defence and adaptive responses [78]. Its variation patterns were strikingly similar in the salt-tolerant *Plantago* taxa, both the halophytes *P. crassifolia* and *P. coronopus* and *P. ovata*, increasing in parallel to the applied NaCl concentration. These data support the notion that H_2_O_2_ is indeed involved in the antioxidant mechanisms of tolerance in salt-tolerant *Plantago* species. On the contrary, in the salt-sensitive *P. afra*, no significant changes in H_2_O_2_ concentrations were detected in salt-treated plants with respect to the non-stressed controls.

Secondary metabolites with antioxidant properties play an important role in the tolerance of plants to salt stress [17]. Among these compounds, particular attention has been given to phenolic compounds and, especially, to the subgroup of flavonoids, because of their strong antioxidant activity [75,79]. It is known that salt stress triggers increased concentrations of phenolic compounds and flavonoids in *Plantago* [43,80] and that the level of antioxidant activity may be related to the degree of salt tolerance, being higher, for example, in the halophyte *P. maritima* in comparison to the salt-sensitive *P. media*, under waterlogging and salinity stresses. Moreover, differences between different species in their phenolic and flavonoid profiles have been proposed as chemotaxonomic markers in this genus [81]. In our experiments, total phenolic compounds and total flavonoids increased in response to the NaCl treatments, in a concentration-dependent manner, in all four *Plantago* species (except for flavonoids in *P. afra*); it is interesting to note that flavonoid levels were higher in *P. ovata* than in the halophytes at all salt concentrations tested in the former species. All these data agree with previous reports that propose *Plantag*o species as a source of bioactive molecules, particularly useful for the prevention of oxidative stress-related diseases, or as functional foods [82,83].

## 4. Material and Methods

### 4.1. Plant Material

This study was conducted on four *Plantago* species, namely *P. coronopus, P. crassifolia, P. ovata* and *P. afra*. The seeds were collected from their natural habitats in grasslands and salt marshes from different contrasted geographical regions in Tunisia.

The corresponding collection sites are listed in Table 3. The geographic locations were recorded by a GPS Model Garmin 72. Seeds were collected, cleaned, dried and stored at 4 °C.

After sterilisation with commercial bleach and several washes with distilled water, the seeds were sown on peat in 1 L pots placed in plastic trays (12 pots per tray). The trays were maintained in a germination chamber under long-day photoperiod (16 h of light), at 23 °C during the day and 17 °C at night, and 50–80% relative humidity. The pots were watered twice per week with deionised water.

### 4.2. Plant Growth, Salt Treatments and Plant Sampling

Salt treatments were started four weeks after sowing. Plants were watered twice a week with solutions of 0 (control), 200, 400, 600 or 800 mM NaCl in deionised water. Each treatment included five individual plants of each species as biological replicas. Plant material (the root and the aerial part of each plant) was harvested after four weeks, and several growth parameters were determined: root length (RL), stem length (SL), and fresh weight of roots (RFW) and leaves (LFW). Part of the fresh root and leaf material was weighed (FW), dried in an oven at 65 °C for ca. 72 h (until constant weight), and weighed again (dry weight, DW) to calculate the water content percentage of roots and leaves, as WC% = [(FW−DW) / FW] × 100.

### 4.3. Electrical Conductivity of the Substrate

The electrical conductivity of the substrate (EC_1:5_) was measured at the end of the treatments. The samples were collected from five pots per species and treatment, and air-dried. Then, a substrate: deionised water (1:5) mix was prepared by stirring at 600 rpm at room temperature. The suspension was filtered through filter paper, and the EC was measured with a Crison 522 conductivity-meter (Crison Instruments, Barcelona, Spain) and expressed in dS m^−1^.

### 4.4. Photosynthetic Pigments Determination

Chlorophyll a (Chl a), chlorophyll b (Chl b) and total carotenoid (Caro) contents were determined as previously described [84]. Fresh leaf material (0.1 g) was ground with liquid nitrogen, one ml of ice-cold 80% acetone was added, and the sample was shaken overnight at 4 °C in the dark. The extract was centrifuged at 13,300×*g*, at 4 °C, the supernatant was collected, and the absorbance was measured at 470, 645 and 663 nm. The following equations were used for the calculation of pigment concentrations, which were finally expressed in mg g^−1^ DW:

Chl a (µg/mL) = 12.21 × (A_663_) − 2.81 × (A_646_)

Chl b (µg/mL) = 20.13 × (A_646_) − 5.03 × (A_663_)

Caro (µg/mL) = (1000 × A_470_ − 3.27 × [Chl a] − 104 × [Chl b])/227

These and all other UV/visible spectrophotometric assays described below were carried out using a UV-1600PC spectrophotometer (VWR, Llinars del Vallès, Barcelona, Spain).

### 4.5. Ion Content Measurements

Concentrations of sodium (Na^+^), potassium (K^+^), and chloride (Cl^−^) were measured in the roots and leaves of plants sampled after the salt treatments, and in the corresponding non-stressed controls, according to Weimberg [85]. Dried material (ca. 0.1 g) was ground to a fine powder and extracted in 15 mL of MilliQ water, incubating the samples for one hour in a water bath, at 95 °C, followed by cooling to room temperature and filtration through a 0.45 μm Gelman nylon filter (Pall Corporation, Port Washington, NY, USA). The cations Na^+^ and K^+^ were quantified with a PFP7 flame photometer (Jenway Inc., Burlington, VT, USA) and the anion using a chlorimeter (Sherwood, model 926, Cambridge, UK).

### 4.6. Proline and Total Soluble Sugars Quantification

Proline (Pro) content was determined in fresh tissue by the ninhydrin-acetic acid method [86]. Free Pro was extracted in 3% aqueous sulphosalicylic acid, and the extract was mixed with acid ninhydrin solution, incubated at 95 °C for 1 h, cooled on ice and then extracted with two volumes of toluene. The absorbance of the organic phase was determined at 520 nm using toluene as a blank. Samples containing known amounts of Pro were assayed in parallel to obtain a standard curve. Pro concentration was expressed as μmol g^−1^ DW.

Total soluble sugars (TSS) were quantified according to Dubois et al. [87]. Fresh leaf material (ca. 0.1 g) was extracted in 3 mL of 80% (*v*/*v*) methanol on a rocker shaker for 24 h. The samples were vortexed and centrifuged at 13,300×*g* for 10 min, and the supernatants were collected and diluted 10-fold with water. The diluted samples were supplemented with concentrated sulphuric acid and 5% phenol, and the absorbance was measured at 490 nm. TSS contents were expressed as ‘mg equivalent of glucose’, used as the standard (mg eq. gluc g^−1^ DW).

### 4.7. Oxidative Stress Markers

Malondialdehyde (MDA) contents were determined following a previously reported procedure [88] with some modifications [89], using the same 80% methanol extracts prepared for TSS quantification. The samples were mixed with 0.5% thiobarbituric acid (TBA) dissolved in 20% trichloroacetic acid (TCA) (or with 20% TCA without TBA for the controls) and then incubated at 95 °C for 20 min. The reactions were stopped on ice, and the samples were centrifuged at 13,300× *g* for 10 min at 4 °C. Finally, the absorbance of the supernatants was determined at 440, 532 and 600 nm. MDA concentration was calculated using the equations previously described [89], based on the molar extinction coefficient at 532 nm of the MDA-TBA adduct (ε_532_ = 155 mM^−1^ cm^−1^).

Measurement of the hydrogen peroxide (H_2_O_2_) content was carried out according to a previously published method [90]. H_2_O_2_ was extracted in a 0.1% (*w*/*v*) TCA solution from 0.1 g fresh leaf material. The extract was centrifuged at 13,300× *g* for 15 min, and the supernatant was collected and mixed with one volume of 10 mM potassium phosphate buffer (pH 7) and two volumes of 1 M KI. Finally, the absorbance of the sample was measured at 390 nm. H_2_O_2_ contents were expressed as μmol g^−1^ DW.

### 4.8. Non-Enzymatic Antioxidants

Total phenolic compounds (TPC) and total flavonoid (TF) contents were measured in the same 80% methanol extracts used for TSS and MDA quantification. TPC were determined as previously described [91] by reaction of the extracts with NaHCO_3_ and the Folin-Ciocalteu reagent. The reaction mixtures were kept in the dark, at room temperature, for 90 min, and the absorbance was then measured at 765 nm. TPC concentration was expressed as equivalents of the gallic acid standard (mg eq. GA g^−1^ DW).

TF were determined by reaction with AlCl_3_ under alkaline conditions after nitration of catechol groups with NaNO_2_ [92]. The absorbance of the samples was read at 510 nm. Catechin was used as a standard to plot a calibration curve, and the results were expressed as catechin equivalents (mg eq. C g^−1^ DW).

### 4.9. Statistical Analysis

Each assay was conducted in a completely randomised design (CRD) with four genotypes and two treatments. Variance analysis was performed to determine the interaction between the different applied treatments and the different species. The measured parameters were subjected to a two-way analysis of variance (ANOVA test). The confidence interval was calculated at the threshold of 95% with mean comparison according to the Tukey test using ‘PLAnt Breeding STATistical software’ (PLABSTAT) [93], version 3A of 2011-06-14. Throughout the text, all values shown are means of five biological replicas (five individual plants) ± standard error (SE).

A Principal Components Analysis (PCA) was carried out on the correlation matrix using PAST software, version 4.03 [94]. The PCA was applied to the data matrix (21 morphological, physiological and biochemical traits × 4 *Plantago* species). The input data contained the mean values of all parameters measured under the different salt stress conditions. The cumulative variability of each parameter was calculated, as well as eigenvalues and principal component scores.

## 5. Conclusions

The four *Plantago* species analysed here can be clearly divided, by several criteria, into two groups: the halophytes *P. crassifolia* and *P. coronopus* and the glycophytes *P. ovata* and *P. afra*. The halophytes, as expected, are highly salt-tolerant, surviving one-month treatment at salinities as high as 800 mM NaCl. Despite not being considered a typical halophyte, *P. ovata* plants are nonetheless relatively resistant to salt, withstanding one month in the presence of 600 mM NaCl. *Plantago afra*, on the other hand, is the most salt-sensitive of the four species, surviving only the 200 mM NaCl treatment.

The most relevant tolerance mechanisms of *P. crassifolia* and *P. coronopus* are based on: (i) the active transport of Na^+^ and Cl^−^ ions to the leaves, where they contribute to cellular osmotic balance under high salinity conditions, as ‘inorganic osmolytes’; (ii) the accumulation of high leaf levels of the organic osmolyte proline; (iii) their relative resistance to the generation of oxidative stress causing membrane lipid peroxidation; and (iv) the salt-induced increase of the levels of antioxidant metabolites, such as phenolic compounds and flavonoids. In *P. ovata*, the efficiency of the above mechanisms is generally lower than in the halophytes, but this limitation is partly compensated by: (i) a more efficient transport to the aerial part and accumulation in the leaves of Cl^−^ ions; (ii) the activation of K^+^ uptake and transport to the leaves under high salinity conditions; (iii) a less pronounced inhibition of photosynthesis, as indicated by the smaller reduction of photosynthetic pigments contents; and (iv) the accumulation of flavonoids in the leaves to relatively higher concentrations than in the halophytes, at salt concentrations of 200 to 600 mM NaCl. Apart from these induced mechanisms, constitutive responses contribute to salt tolerance in the three species, namely the accumulation in leaves of inorganic ions at high concentrations in control, non-stressed plants. Summarising, *P. ovata*, not considered a halophytic species, is nevertheless quite resistant to salt stress but using tolerance mechanisms somewhat different from those of the typical congeneric halophytes, *P. crassifolia* and *P. coronopus*.

This work confirms the usefulness of performing comparative studies on the responses to stress of taxonomically related species with different degrees of resistance to the particular stressful condition, to identify the most relevant tolerance mechanisms.

## Figures and Tables

**Table 1:** Results of two-way ANOVA (F ratios) for the independent factors ‘Species’ (S), ‘Treatment’ (T) and the interaction ‘Species × Treatment’ (S × T). Abbr.: ECs: electrical conductivity of the substrate; RL: root length; SL: shoot length; FW(%): leaf fresh weight (as a percentage of the control); RWC: root water content; LWC: leaf water content; Chl a: Chlorophyll a; Chl b: Chlorophyll b; Caro: carotenoids; Na^+^ R: sodium in roots; Na^+^ L: sodium in leaves; Cl^−^ R: chloride in roots; Cl^−^ L: chloride in leaves; K^+^ R: potassium in roots; K^+^ L: potassium in leaves; MDA: malondialdehyde; H_2_O_2_: hydrogen peroxide; TSS: total soluble sugars; Pro: proline; TPC: total phenolic compounds; TF: total flavonoids.

Variables	S	T	S × T
ECs	297.04 *	129.78 *	62.07 *
RL	205.23 *	34.43 *	70.59 *
SL	35.99 *	53.34 *	8.65 *
FW (%)	10.29 *	66.62 *	1.56
RWC	91.76 *	49.30 *	17.78 *
LWC	142.33 *	87.91 *	16.21 *
Chl a	17.68 *	45.54 *	8.04 *
Chl b	11.69 *	29.51 *	2.68 *
Caro	12.59 *	48.68 *	3.30 *
Na^+^ R	239.91 *	26.67 *	22.49 *
Na^+^ L	235.82 *	62.31 *	49.70 *
Cl^−^ R	23.76 *	10.69 *	7.04 *
Cl^−^ L	77.36 *	23.50 *	29.56 *
K^+^ R	40.97 *	21.01 *	3.45 *
K^+^ L	63.60 *	0.90	9.12 *
MDA	55.67 *	102.62 *	29.84 *
H_2_O_2_	102.17 *	11.32 *	22.67 *
TSS	7.75 *	58.23 *	20.62 *
Pro	192.94 *	63.45 *	33.07 *
TPC	40.68 *	10.35 *	24.94 *
TF	93.51 *	18.10 *	51.53 *

* Significant at the 95% confidence level.

**Table 2:** Electrical conductivity of the substrate in 1:5 soil:water suspensions (EC_1:5_, dS m^−1^), in pots watered for four weeks with the indicated NaCl concentrations. The values shown are means ± SE (*n* = 5). In each row, different letters indicate significant differences between treatments, according to the Tukey test (*p* < 0.5). n.d.: not determined.

	Control	200 mM	400 mM	600 mM	800 mM NaCl
*P. crassifolia*	0.48 ± 0.02 ^a^	3.56 ± 0.18 ^b^	5.46 ± 0.22 ^c^	7.94 ± 0.50 ^d^	9.45 ± 0.30 ^e^
*P. coronopus*	0.45 ± 0.02 ^a^	1.20 ± 0.09 ^b^	5.66 ± 0.50 ^c^	7.60 ± 0.10 ^d^	9.57 ± 0.20 ^e^
*P. ovata*	0.45 ± 0.02 ^a^	1.51 ± 0.05 ^b^	5.63 ± 0.14 ^c^	8.51 ± 0.40 ^d^	n.d.
*P. afra*	0.49 ± 0.05 ^a^	2.15 ± 0.15 ^b^	n.d.	n.d.	n.d.

**Table 3:** Origin and bioclimatic zones of the studied *Plantago* species.

Species	Subgenus	Location	Latitude	Longitude	Bioclimatic Zones
*P. coronopus* L.	*Coronopus*	Hergla/Sousse	35°58′52.07″	10°31′38.14″	Semi-arid inferior
*P. crassifolia* Forsk.	*Coronopus*	Djerba/Mednine	33°49′52.96″	11°2′17.67″	Arid inferior
*P. ovata* Forsk.	*Psyllium*	Tataouine	32°55′8.63″	10°24′59.45″	Saharian superior
*P. afra* L.	*Psyllium*	Bouargoub	36°28′34.84″	10°36′46.35″	Semi-arid superior

## Data Availability

Data are contained within the article.
